# Application of extracorporeal membrane oxygenation in patients with severe acute respiratory distress syndrome induced by avian influenza A (H7N9) viral pneumonia: national data from the Chinese multicentre collaboration

**DOI:** 10.1186/s12879-017-2903-x

**Published:** 2018-01-08

**Authors:** Linna Huang, Wei Zhang, Yi Yang, Wenjuan Wu, Weihua Lu, Han Xue, Hongsheng Zhao, Yunfu Wu, Jia Shang, Lihua Cai, Long Liu, Donglin Liu, Yeming Wang, Bin Cao, Qingyuan Zhan, Chen Wang

**Affiliations:** 1 Department of Pulmonary and Critical Care Medicine, Centre for Respiratory Diseases, China-Japan Friendship Hospital, No. 2 Yinghua East Road, Chaoyang District, Beijing, 100029 People’s Republic of China; 2 The First Affiliated Hospital of Nanchang University, Nanchang, Jiangxi Province People’s Republic of China; 3 Department of Critical Care Medicine, Zhongda Hospital, Southeast University, Nanjing, Jiangsu Province People’s Republic of China; 4 Department of Intensive Care Unit, Wuhan Medical Treatment Center Hospital, Wuhan, Hubei Province People’s Republic of China; 5 Department of Intensive Care Unit, The First Affiliated Hospital of Wannan Medical College, Yijishan Hospital, Wuhu, Anhui Province People’s Republic of China; 6 Department of Intensive Care Unit, Taizhou People’s Hospital, Taizhou, Jiangsu Province People’s Republic of China; 7 Department of Intensive Care Unit, Affiliated Hospital of Nantong University, Nantong, Jiangsu Province People’s Republic of China; 8 Department of Intensive Care Unit, Suzhou Municipal Hospital, Suzhou, Jiangsu Province People’s Republic of China; 9 Department of Infectious Diseases, Henan Provincial People’s Hospital, Zhengzhou, Henan Province People’s Republic of China; 10 Department of Intensive Care Unit, Dongguan People’s Hospital, Dongguan, Guangdong Province People’s Republic of China; 11 Department of Intensive Care Unit, The First People’s Hospital of Kunshan, Kunshan, Jiangsu Province People’s Republic of China

**Keywords:** Extracorporeal membrane oxygenation (ECMO), Avian influenza A (H7N9), Acute respiratory distress syndrome (ARDS), Complications, Mortality

## Abstract

**Background:**

Evidence concerning the efficacy and safety of extracorporeal membrane oxygenation (ECMO) in patients with influenza A (H7N9) has been was limited to case reports. Our study is aimed to investigate the current application, efficacy and safety of ECMO in for severe H7N9 pneumonia-associated acute respiratory distress syndrome (ARDS) in the Chinese population.

**Methods:**

A multicentre retrospective cohort study was conducted at 20 hospitals that admitted patients with avian influenza A (H7N9) viral pneumonia patients’ admission from 9 provinces in China between October 1, 2016, and March 1, 2017. Data from the National Health and Family Planning Commission of China, including general conditions, outcomes and ECMO management, were analysed. Then, successfully weaned and unsuccessfully weaned groups were compared.

**Results:**

A total of 35 patients, aged 57 ± 1 years, were analysed; 65.7% of patients were male with 63% mortality. All patients underwent invasive positive pressure ventilation (IPPV), and rescue ventilation strategies were implemented for 23 cases (65.7%) with an average IPPV duration of 5 ± 1 d, PaO_2_/FiO_2_ of 78 ± 23 mmHg, tidal volume (VT) of 439 ± 61 ml and plateau pressure (P_plat_) of 29 ± 8 cmH_2_O pre-ECMO. After 48 h on ECMO, PaO_2_ improved from 56 ± 21 mmHg to 90 ± 24 mmHg and PaCO_2_ declined from 52 ± 24 mmHg to 38 ± 24 mmHg. Haemorrhage, ventilator-associated pneumonia (VAP) and barotrauma occurred in 45.7%, 60% and 8.6% of patients, respectively. Compared with successfully weaned patients (*n* = 14), the 21 unsuccessfully weaned patients had a longer duration of IPPV pre-ECMO (6 ± 4 d vs. 2 ± 1 d, *P* < 0.01) as well as a higher P_plat_ (25 ± 5 cmH_2_O vs. 21 ± 3 cmH_2_O, *P* < 0.05) and VT (343 ± 96 ml vs. 246 ± 93 ml, P < 0.05) after 48 h on ECMO support. Furthermore, the unsuccessfully weaned group had a higher mortality (100% vs. 7.1%, P < 0.01) with more haemorrhage (77.3% vs. 28.6%, *P* < 0.01).

**Conclusions:**

ECMO is effective at improving oxygenation and ventilation of patients with avian influenza A (H7N9) induced severe ARDS. Early initiation of ECMO with appropriate IPPV settings and anticoagulation strategies are necessary to reduce complications.

## Background

Avian influenza A (H7N9) viral pneumonia can manifest with varying degrees of dyspnea and is associated with a mortality of ~30% [1]. In particular, 97% of patients develop rapidly progressive pneumonia and 71% progress to acute respiratory distress syndrome (ARDS). The mortality of severe ARDS is as high as 62% [2].

Timely and effective respiratory support is particularly important to treat severe ARDS caused by avian influenza A (H7N9) pneumonia. However, severe ARDS induced by avian influenza A (H7N9) pneumonia might manifest as refractory hypoxaemia even with appropriate invasive positive pressure ventilation (IPPV) support. Extracorporeal membrane oxygenation (ECMO) is the ultimate respiratory support method and directly improves the oxygenation and ventilation of patients as well as enables implementation of the “lung protective ventilation strategy” [3]. ECMO was the breakthrough treatment for the severe avian influenza A (H1N1) outbreak of 2009 and reduced mortality from this outbreak [4–6]. Therefore, we believe that ECMO could also be effective for other types of severe viral pneumonia.

Existing studies of ECMO treatment for avian influenza A (H7N9) pneumonia are primarily limited to case reports [7–9], and no study has systematically reviewed the efficacy or safety of ECMO to treat such diseases. Therefore, it is particularly important to understand the current application of ECMO for avian influenza A (H7N9) pneumonia-induced severe ARDS, investigate the application timing and management strategies of ECMO, and explore the possible reasons for treatment failure. Based on the current study, we expect to standardize the management of ECMO and provide a description of our experiences using ECMO to treat patients with avian influenza A (H7N9) pneumonia-induced severe ARDS.

## Methods

### Study population and data collection

Patients who had laboratory-confirmed avian influenza A (H7N9) virus-induced pneumonia were included in this study. Patients were admitted to 20 hospitals in 9 provinces of China between October 1, 2016, and March 1, 2017, and were reported to the National Health and Family Planning Commission of China.

We included patients aged >14 ys who were supported by ECMO. Patients who were lacking key detailed records of parameters during ECMO, such as ventilator or laboratory findings, were excluded.

The included patients were divided into 2 groups, namely, the “successfully weaned group” and “unsuccessfully weaned group”. The former refers to a group of patients whose condition improved and were weaned from ECMO for at least 48 h; the “unsuccessfully weaned group” refers to those who died or voluntarily discontinued treatment due to lack of improvement during ECMO support.

#### General conditions

The general conditions included age, gender, pregnancy status, underlying disease, time from onset to antiviral drug administration, vasoactive drug administration pre-ECMO, duration of IPPV pre-ECMO, whether rescue ventilation strategies (including lung recruitment maneuvre, prone-position ventilation, and high-frequency oscillation ventilation) were implemented pre-ECMO, disease severity score, total duration of ECMO and IPPV.

#### Conditions during ECMO

We collected the ECMO blood flow at 24, 48, 72, and 96 h on ECMO. Improvement in circulatory and respiratory physiological indicators were considered, as well as IPPV parameters at 6 h pre-ECMO and 24, 48, and 72 h on ECMO. Furthermore, anticoagulation indicators during ECMO, including the types of anticoagulant drugs and methods of use; the maximum and minimum values of the activated coagulation time (ACT) and activated partial thromboplastin time (APTT); and the differences between the maximum and minimum ACT and APTT at 24, 48, and 72 h on ECMO were recorded. Finally, data regarding complications during ECMO therapy, including ECMO and IPPV-related complications and nosocomial infections, were collected.

### Study definitions and design

#### Outcomes

The primary outcome was in-hospital mortality. The secondary outcomes were the length of stay in the intensive care unit (ICU) and total length of hospitalization.

#### Definition of avian influenza A (H7N9) virus infection

Three methods were used for a laboratory diagnosis, namely, the real-time reverse transcription-polymerase chain reaction (RT-PCR), viral isolation, and serological testing for the avian influenza A (H7N9) virus using a modified haemagglutinin inhibition assay [10–12].

#### Definition of acute respiratory syndrome (ARDS)

We defined ARDS according to the Berlin definition in 2012 [13, 14].

#### Definitions of pneumonia and severe pneumonia

Pneumonia was diagnosed as an acute illness with fever, cough, or dyspnea/tachypnea, and at least one new focal chest sign that was supported by a finding of lung shadowing on a chest radiograph and without other non-infectious causes.

The primary criteria for severe pneumonia were as follows: <1 > need for tracheal intubation and mechanical ventilation (MV) and <2 > need for vasoactive drugs after the active fluid resuscitation due to septic shock. The secondary criteria were as follows: <1 > respiratory rate ≥ 30 times/min; <2 > PaO_2_/FiO_2_ ≤ 250 mmHg; <3 > multiple lobe infiltration; <4 > disturbances of consciousness, disorientation, or both; <5 > blood urea nitrogen ≥ 7.14 mmol/L; and <6 > systolic blood pressure ≤ 90 mmHg that required active fluid resuscitation. Patients who met one primary criterion or at least three secondary criteria were diagnosed as having severe pneumonia [15].

#### Definitions of ventilator-associated pneumonia (VAP)

The criteria for the diagnosis of VAP are in accordance with the European Centre for Disease Prevention and Control [16] and included the following: <1 > two or more sequential chest x-rays or CT scans with a suggestive image of pneumonia for patients with underlying cardiac or pulmonary disease, or one definitive chest x-ray or CT scan in patients without underlying cardiac or pulmonary disease; <2 > a fever greater than 38 °C and/or leukocytosis greater than or equal to 12,000 WBC/mm^3^ or leukopenia less than or equal to 4000 WBC/mm^3^; and <3 > at least one of the following: <a > new onset of purulent sputum or change in the characteristics of the sputum; <b > cough, dyspnea, or tachycardia; <c > auscultatory findings, such as rales, bronchial breath sounds, ronchi, or wheezing; or <d > worsening gas exchange (e.g., oxygen desaturation or increased oxygen requirements or increased ventilation demand).

For all included patients, we first described the general conditions, ECMO model and parameters, IPPV parameters, the changes in circulation and respiratory physiological indicators from pre-ECMO to on ECMO status, anticoagulation on ECMO, and complications during ECMO therapy in all included patients. Then, we compared patients who were successfully or unsuccessfully weaned from ECMO with regard to above items.

### Statistical analysis

All of the analyses were performed using SPSS 17.0 software. Normally distributed continuous variables are expressed as the means ± SD and were compared using the t-test or chi-square test. Non-normally distributed continuous variables are expressed as medians and quartiles and were compared using the Wilcoxon rank-sum test. Categorical variables were compared using the *x*^2^ test. *P*-values < 0.05 were considered significant.

## Results

**Fig. 1:**
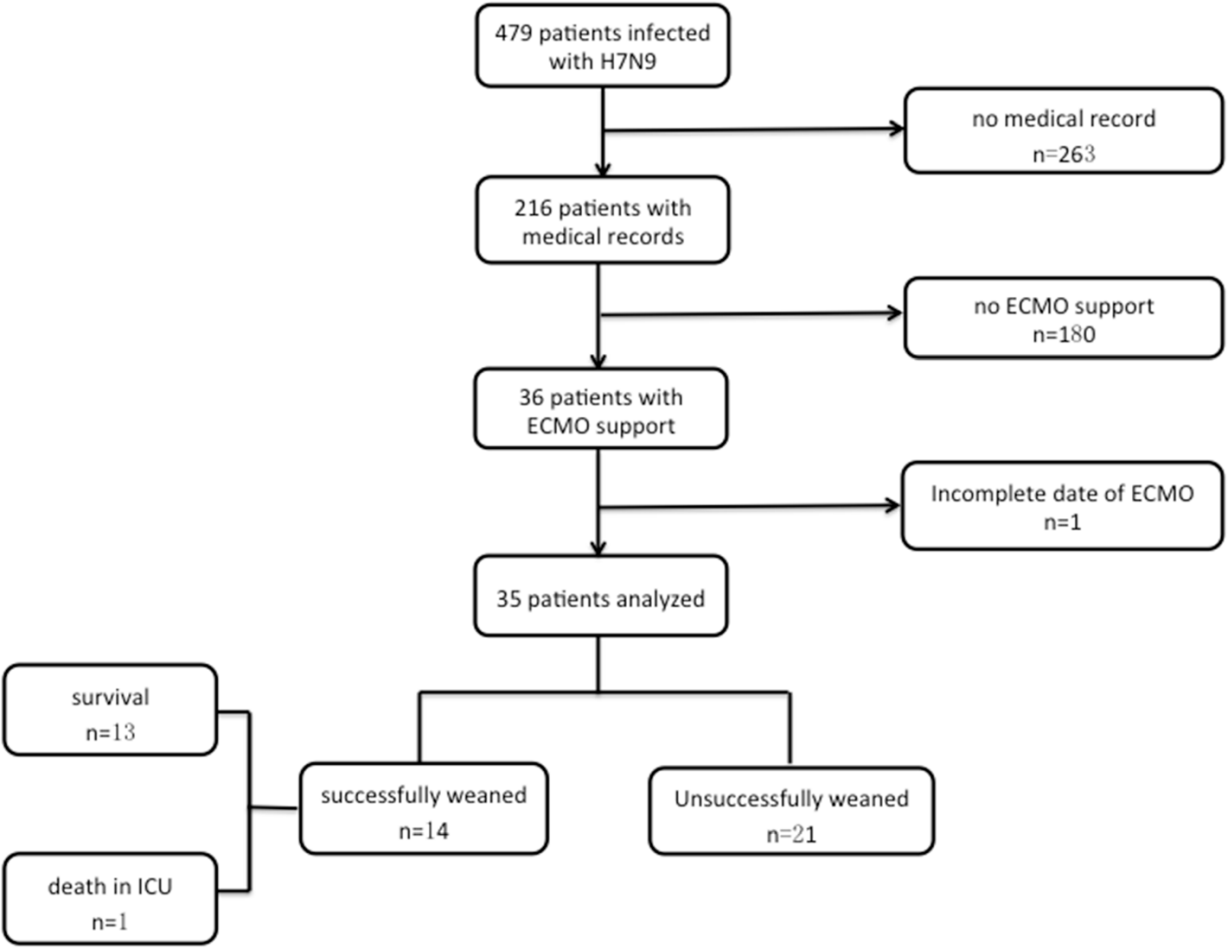
Flowchart. A flowchart illustrating the enrolment of patients with avian influenza A (H7N9) virus-induced pneumonia who were reported to the National Health and Family Planning Commission of China; patients were admitted to 20 hospitals in 9 provinces of China between October 1, 2016, and March 1, 2017

A total of 473 patients were diagnosed with avian influenza A (H7N9) virus-related pneumonia. Patients were admitted to 20 hospitals in 9 provinces of China between October 1, 2016, and March 1, 2017, and were reported to the National Health and Family Planning Commission of China. The medical records of 216 patients were available, and 36 patients were reported to be supported by ECMO. One of the 36 patients lacked IPPV and ECMO parameters pre-ECMO and on ECMO and was eliminated as a participant; therefore, 35 patients were ultimately selected (Fig. 1).

### General conditions and outcomes

Data from 35 patients (65.7% males), with an average age of 57 ± 1 years, were analysed. There was no patient under the age of 16. A total of 22 patients had underlying diseases, 8 patients were treated with steroids and immunosuppressive agents within 1 month of admission to the hospital, and 1 patient was pregnant. The Sequential Organ Failure Assessment (SOFA) score was 9 ± 3 points, and the Murray score was 3.6 ± 0.4 points. The time from onset to antiviral drug administration was approximately 9 ± 5 d, and the time from onset to ECMO support was approximately 10 ± 3 d. High-dose vasoactive drugs [17] were needed to maintain blood pressure in 20 patients (57.1%). The duration of IPPV pre-ECMO was approximately 5 ± 1 d. Rescue ventilation strategies, including the recruitment manoeuvre (RM), prone-position ventilation (PP) and high frequency oscillatory ventilation (HFOV), were needed for 23 patients (65.7%) pre-ECMO. The total durations of IPPV and ECMO were approximately 20 ± 8 d and 8 d (5–13 d), respectively. Of the 35 patients, 14 (40%) were successfully weaned from ECMO, and the other 21 patients died due to an uncontrolled haemorrhage (5 patients), septic shock (6 patients due to progressive lung infection, 3 patients due to bloodstream infection), heart failure (2 patients) and discontinuation of treatment because of no improvement (5 patients). One of 14 patients showed an aggregated lung infection after weaning and eventually died due to septic shock. The in-hospital mortality was 63%. The length of ICU stay was 27 ± 14 d, and the total length of hospitalization was 31 ± 14 d (Table 1).

**Table 1:** General conditions and outcomes of patients with ECMO therapy

Variable	Total (*n* = 35)	Successfully weaned group (*n* = 14)	Unsuccessfully weaned group (*n* = 21)	*P* value
Gender, number (%)				
Male	23(65.7)	9(64.3)	14(66.7)	1.00
Age, years, mean ± SD	57 ± 1	51 ± 10	60 ± 12	0.02*
Underlying Diseases, number (%)				
Hypertension	19(54.3)	7(50)	12(57.1)	0.74
Diabetes	13(37.1)	1(7.1)	12(57.1)	< 0.01*
Cerebrovascular disease	5(14.3)	3(21.4)	2(9.5)	0.37
History of steroid and immunosuppressant within 1 month	8(22.9)	4(28.6)	4(19.0)	0.69
Pregnancy, number (%)	1(2.9)	0(0.0)	1(47.6)	1.00
Onset to NAI	9 ± 5	7 ± 3	11 ± 6	0.04*
Onset to ECMO	10 ± 3	10 ± 3	10 ± 3	0.97
Organ support pre-ECMO				
High-dose vasoactive drugs^a^, number (%)	20(57.1)	6(42.9)	14(63.6)	0.31
Rescue ventilation strategies^b^, number (%)	23(65.7)	6(43.9)	17(81.0)	0.03*
Duration of IPPV, days, mean ± SD	5 ± 1	2 ± 1	6 ± 4	< 0.01*
SOFA Score	9 ± 3	7 ± 2	10 ± 4	0.02*
Murray Score	3.6 ± 0.4	3.6 ± 0.5	3.6 ± 0.4	0.95
PaO_2_/FiO_2_ pre-ECMO, mmHg, mean ± SD	78 ± 23	89 ± 24	71 ± 20	0.04*
Duration of ECMO, days, median (IQR)	8(5–13)	10(7–13)	7(3–16)	0.48
Total Duration of IPPV, days, mean ± SD	20 ± 8	23 ± 13	18 ± 15	0.36
Clinical outcomes				
Mortality, number (%)	22 (63)	1 (7.1)	21 (100)	< 0.01*
Length of stay in ICU, days, mean ± SD	27 ± 14	33 ± 12	24 ± 14	0.081
Length of hospitalization, days, mean ± SD	31 ± 14	39 ± 10	27 ± 14	0.010*

Note: ^a^high-dose vasoactive drugs [17] infer to: noradrenaline > 0.5 μg/kg/min or dopamine > 20 μg/kg/min; ^b^rescue ventilation strategies includes recruitment maneuver (RM), prone position ventilation (PP) and high frequency oscillatory ventilation (HFOV)

*NAI* neuraminidase inhibitors, *IPPV* invasive positive pressure ventilation, *ECMO* extracorporeal membrane oxygenation, *SOFA score* sequential organ failure score, *ICU* intensive care unit

We made comparison between successfully weaned and unsuccessfully weaned group using t-test, x^2^ test or Wilcoxon rank-sum test, and *P* values <0.05 were considered significant, marked as *

### Conditions during ECMO

#### ECMO mode and information concerning the equipment

Of the 35 patients, 33 were treated using the veno-venous ECMO (V-V ECMO) model. A total of 2 patients with severe cardiac insufficiency and cardiogenic shock were treated using the venous-arterial ECMO (V-A ECMO) model. The ECMO equipment was mainly provided by MAQUET (Shanghai) Medical Equipment Co., Ltd. and SORIN (Shanghai) Medical Equipment Co., Ltd. The pump from SORIN was the Stockert Centrifugal Pump System (SCP/SCPC), and the oxygenator was the D905 EOS ECMO. The pump from MAQUET was the ROTAFLOW, and the oxygenator was the QUADROX PLS.

#### ECMO management

##### Changes in IPPV parameters and physiological indicators in patients on ECMO

The ventilator parameters, including FiO_2_, positive end-expiratory pressure (PEEP), P_plat_, and VT, were significantly decreased in patients on ECMO. The vital signs, which included the heart rate, respiratory rate, and SpO_2,_ and the arterial blood gas analysis (ABG), which included the pH, PaCO_2_, and PaO_2_ levels, were improved in patients after ECMO support (Table 2).

**Table 2:** Parameters on ECMO, IPPV parameters and physiological indicators pre and on ECMO

Variable	6 h pre-ECMO	24 h on ECMO	48 h on ECMO	72 h on ECMO	*P* value
Blood flow (L/min)	–	3.84 ± 0.87	4.48 ± 0.77	4.18 ± 1.00	0.023*
IPPV parameters					
FiO_2_	94 (55–100)	50 (40–80)	45 (40–60)	46 (40–50)	<0.001*
PEEP (cmH_2_O)	14 ± 4	12 ± 5	11 ± 3	11 ± 2	0.022*
VT (ml)	439 ± 61	285 ± 94	317 ± 103	308 ± 128	<0.001*
P_plat_ (cmH_2_O)	29 ± 8	23 ± 6	23 ± 5	24 ± 7	0.004*
Physiological indicators					
Heart Rate (/min)	103 ± 27	90 ± 24	85 ± 22	90 ± 25	0.025*
MAP (mmHg)	84 ± 15	87 ± 15	81 ± 21	94 ± 17	0.057
Respiratory Rate (/min)	31 ± 7	19 ± 6	16 ± 6	16 ± 6	<0.001*
pH	7.33 ± 0.10	7.42 ± 0.10	7.39 ± 0.10	7.43 ± 0.10	0.006*
PaCO_2_ (mmHg)	52.4 ± 16.6	34.1 ± 9.8	38.3 ± 10.5	38.2 ± 7.8	<0.001*
PaO_2_ (mmHg)	56.3 ± 20.9	90.0 ± 35.9	94.8 ± 45.7	104.4 ± 57.5	<0.001*
Lactate (mmol/L)	2.8 ± 1.9	3.1 ± 3.7	4.0 ± 4.8	2.9 ± 2.7	0.625

Note: *ECMO* extracorporeal membrane oxygenation, *IPPV* invasive positive pressure ventilation, *FiO*_*2*_ fraction of inspiration, *PEEP* positive end-expiratory pressure, *VT* tidal volume, *P*_*plat*_ plateau pressure, *MAP* mean arterial pressure, *SpO*_*2*_ fingertip pulse oxygen saturation, *PaCO*_*2*_ partial pressure of arterial carbon dioxide, *PaO*_*2*_ partial pressure of arterial oxygen

*P < 0.05

##### Monitoring of anticoagulation

All 35 patients received a continuous infusion of unfractionated heparin for anticoagulation. However, heparin was discontinued for 2 patients with cerebral haemorrhage and 3 with active gastrointestinal haemorrhage. The ACT was 144 ± 61 to 220 ± 66 s at 24 h, 16 ± 37 to 234 ± 37 s at 48 h, and 147 ± 26 to 221 ± 40 s at 72 h on ECMO. The APTT was 58 ± 23 to 84 ± 28 s and 53 ± 15 to 75 ± 23 s at 48 and 72 h on ECMO, respectively.

##### Complications during ECMO therapy

In this study, the rates of gastrointestinal haemorrhage, cerebral haemorrhage, brain death, renal insufficiency, disseminated intravascular coagulation (DIC), hyperglycaemia, and ECMO oxygenator thrombosis were higher compared to the relevant data from the *ECLS Registry Report* [18, 19]. New cases of VAP developed in 21 patients during ECMO, with an incidence rate of 60%. New cases of barotrauma occurred in 3 patients, accounting for 8.6% of cases. In addition, 6 patients had a urinary infection, with an incidence rate of 17.1%, and 10 patients had a catheter-related blood stream infection (CRBSI), with an incidence rate of 28.6% (Table 3).

**Table 3:** Complications During ECMO

Complications	Our study (%)	ECLS Registry Report (%) [18, 19]
ECMO Mechanical Complications		
Oxygenator failure	0.0	18
Oxygenator thrombosis	14.3	12
Other sites thrombosis	2.9	7.7
Hemorrhage	45.7	–
Gastrointestinal hemorrhage	28.6	4.6
Cerebral hemorrhage	8.6	4–8
Other site hemorrhage	31.4	–
Organ Failure		
Brain death	8.6	3.8
Cerebral infarction	2.9	–
Epilepsy	2.9	–
Renal insufficiency	48.6	33.5
Heart failure	62.9	61.8
Arrhythmia with unstable hemodynamics	5.7	18.2
Cardiac arrest	11.4	9.8
Liver failure	25.7	–
DIC	8.6	3.8
Hemolysis	2.9	7.1
Severe thrombocytopenia	11.4	–
Nosocomial Infection		
CRBSI	28.6	21.2
Bacteremia of other sources	5.7	–
VAP	60.0	–
Urinary infections	17.1	–
Barotrauma	8.6	–
Metabolic		
Hyperbilirubinemia	4 (11.4)	7.3
Hyperglycemia	17 (48.6)	18.2

Note: *DIC* disseminated intravascular coagulation, *CRBSI* catheter related bloodstream infection, *VAP* ventilator associated pneumonia; severe thrombocytopenia refers to thrombocyte less than 20*10^9/L; hyperglycemia refers to blood glucose more than 13.3 mmol/L

### Comparison between the patients successfully and unsuccessfully weaned from ECMO

Group 1 contained 14 patients who were successfully weaned from ECMO, and group 2 included 21 patients who were unsuccessfully weaned from ECMO.

Compared with patients successfully weaned from ECMO, the unsuccessfully weaned group had a higher mortality (100% vs. 7.1%, respectively, *P* < 0.01), and was older (60 ± 12 years vs. 51 ± 10 years, respectively, *P* = 0.02), and more likely to have diabetes mellitus (57.1% vs. 7.1%, respectively, *P* < 0.01), had more frequent severe conditions (SOFA: 10 ± 4 points vs. 7 ± 2 points, respectively, P < 0.01) pre-ECMO. Meanwhile, they had a longer duration of IPPV (6 ± 4 d vs. 2 ± 1 d, respectively, *P* < 0.01), had lower PaO_2_/FiO_2_ levels (71.4 ± 20.0 mmHg vs. 89.3 ± 24.2 mmHg, respectively, *P* < 0.05), and higher rate of rescue ventilation strategies (81% vs. 43.9%, respectively, *P* < 0.05) before ECMO support. No significant differences were found in the total duration of IPPV, total duration of ECMO, length of ICU stay and length of hospitalization between the two groups (Table 1).

#### Blood flow in patients on ECMO

ECMO blood flow did not significantly differ between the two groups during the initiation of ECMO support. However, in the successfully weaned group vs. the unsuccessfully weaned group, a significant decrease in blood flow correlated with an increase in the duration of support, which was 3.65 ± 0.70 L/min vs. 4.57 ± 1.02 L/min, respectively, (*P* < 0.05) at 72 h on ECMO and 3.65 ± 0.86 L/min vs. 4.62 ± 0.90 L/min, respectively, (*P* < 0.01) at 96 h on ECMO (Additional files 1 and 2).

#### IPPV parameters in patients on ECMO

**Fig. 2:**
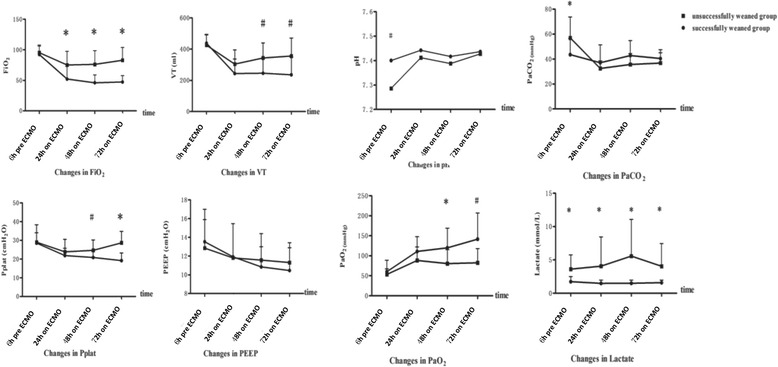
Comparison of IPPV parameters and ABGs between two groups of patients on ECMO. For the successfully weaned group compared to the unsuccessfully weaned group, FiO_2_ was 46 ± 13% vs. 74 ± 25%, respectively, at 48 h (*P* < 0.01) and 45 ± 11% vs. 78 ± 24%, respectively, at 72 h (*P* < 0.01). The monitored P_plat_ was 21 ± 3 cmH_2_O vs. 25 ± 5 cmH_2_O, respectively, at 48 h (*P* < 0.05) and 19 ± 4 cmH_2_O vs. 29 ± 6 cmH_2_O, respectively, at 72 h (P < 0.01). The monitored VT was 246 ± 93 ml vs. 343 ± 96 ml, respectively, at 48 h (P < 0.05) and 236 ± 113 ml vs. 356 ± 116 ml, respectively, at 72 h (P < 0.05) after ECMO support. However, there were no differences in PEEP during ECMO between the two groups. Patients who were in the unsuccessfully weaned group compared to patients in the successfully weaned group had severe acidosis (pH: 7.29 ± 0.14 vs. 7.40 ± 0.05, respectively, (P < 0.01), a higher PaCO_2_ (57.0 ± 16.7 mmHg vs. 43.6 ± 13.0 mmHg, respectively, (P < 0.05), and a higher lactate concentration (3.6 ± 2.1 mmol/L vs. 1.7 ± 0.8 mmol/L, respectively (P < 0.05), pre-ECMO. The pH and PaCO_2_ did not significantly differ between the two groups during ECMO therapy, while patients who eventually weaned successfully from ECMO had a gradual ascending tendency in PaO_2_ at 48 and 72 h on ECMO and a sustained low level of lactate after ECMO therapy

In the successfully weaned group compared to the unsuccessfully weaned group, FiO_2_ was 46 ± 13% vs. 74 ± 25%, respectively, (*P* < 0.01) at 48 h and 45 ± 11% vs. 78 ± 24%, respectively, (*P* < 0.01) at 72 h on ECMO; monitored P_plat_ was 21 ± 3 cmH_2_O vs. 25 ± 5 cmH_2_O, respectively, (*P* < 0.05) at 48 h, and 19 ± 4 cmH_2_O vs. 29 ± 6 cmH_2_O, respectively, (P < 0.01) at 72 h on ECMO; and monitored VT was 246 ± 93 ml vs. 343 ± 96 ml, respectively, (*P* < 0.05) at 48 h, and 236 ± 113 ml vs. 356 ± 116 ml, respectively, (*P* < 0.05) at 72 h on ECMO; all of the differences were statistically significant (Fig. 2, Additional file 1).

#### Improvement in circulatory and respiratory physiological indicators on ECMO

The vital signs were improved but did not significantly differ between the two groups pre-ECMO and during ECMO support (Additional file 3). Patients who were unsuccessfully weaned from ECMO compared to patients who were successfully weaned from ECMO had severe acidosis (pH: 7.29 ± 0.14 vs. 7.40 ± 0.05, respectively, (*P* < 0.01), a higher PaCO_2_ (57.0 ± 16.7 mmHg vs. 43.6 ± 13.0 mmHg, respectively, (*P* < 0.05), and a higher lactate concentration (3.6 ± 2.1 mmol/L vs. 1.7 ± 0.8 mmol/L, P < 0.05) pre-ECMO. pH and PaCO_2_ did not differ significantly between the two groups during ECMO support, while patients who were eventually successfully weaned from ECMO had a gradual ascending tendency of PaO_2_ at 48 and 72 h on ECMO and a sustained low level of lactate (Fig. 2, Additional file 1).

#### Changes in anticoagulation on ECMO

During the early stage of ECMO (24 and 48 h), the successful weaning group showed smaller differences between the ACT_max_ and ACT_min_ than the unsuccessful weaning group, which was 48 ± 20 s vs. 104 ± 35 s at 24 h (*P* < 0.05) and 40 ± 25 s vs. 98 ± 40 s at 48 h (*P* < 0.01). However, this trend was not found with regard to the difference between the maximum and minimum APTT.

#### Complications during ECMO

There were no differences between the two groups in mechanical complications associated with ECMO, VAP and barotrauma. The successfully weaned group compared to the unsuccessfully weaned group had a lower haemorrhage rate (28.6% vs. 77.3%, respectively, *P* < 0.01), lower rate of renal insufficiency (21.4% vs. 63.6%, respectively, *P* < 0.05), lower rate of liver failure (0% vs. 40.9%, respectively, *P* < 0.01) and lower heart failure rate (35.7% vs. 77.3%, respectively, *P* < 0.05).

## Discussion

This study was the first to systematically and comprehensively discuss as well as elaborate on the current application of the efficacy and safety of ECMO in patients with H7N9 pneumonia-related ARDS.

A few studies [17, 20, 21, 22] have shown that the mortality of pH1N1-induced ARDS was reduced to 21–61% following ECMO treatment. Presently, no studies with large samples have investigated the mortality of H7N9-induced ARDS, while the in-hospital mortality was as high as 63% in our study. Late initiation of ECMO, inappropriate IPPV settings during ECMO, and more ECMO complications might explain the relatively high mortality. Moreover, as a multicentre collaboration study, the experiences of ECMO varied among the centres (Additional file 4), which might be another reason for the high mortality.

According to the Extracorporeal Life Support Organization (ELSO) data [23, 24], ECMO is indicated when death risk exceeds 80%, i.e., when PaO_2_/FiO_2_ < 80 mmHg on FiO_2_ > 90% and the Murray score is 3–4. Our patients met the indications for ECMO support. The duration of MV for more than 7 days pre-ECMO is an important prognostic factor for death [25]. For patients in the successfully weaned group, the duration of IPPV pre-ECMO was 5 ± 1 d; however, the duration was even longer among patients in the unsuccessfully weaned group (6 ± 4 d). Moreover, rescue ventilation strategies were implemented for most patients before ECMO, which partially delayed the timing of ECMO. In comparison, ECMO was initiated at 2 h (1–5 h) after IPPV among patients with pH1N1 in Australia and New Zealand in 2009 [5], which was significantly shorter than that in our cases. Therefore, we emphasized early implementation of ECMO in our patients.

The principle of IPPV during ECMO is the “lung rest strategy” [26]. The REVA registry study examined 123 patients with pH1N1-induced ARDS [6] and showed that the high P_plat_ (29 cmH_2_O) on day 1 of ECMO was related to high mortality. In our study, the pre-ECMO P_plat_ level was high (29 ± 8 cmH_2_O). High P_plat_ can lead to overdistension of the alveoli and cause lung volutrauma. The shear force between the overdistended and collapsed alveoli further aggregates VILI [27], which ultimately increases mortality. Although the P_plat_ values decreased to different degrees after ECMO, the P_plat_ of the unsuccessfully weaned group was significantly higher at 48 and 72 h during ECMO. The principle of low VT was similar in that we found a lower VT during ECMO in the successfully weaned group. A retrospective observational study of 168 patients with severe ARDS [28] showed that a high PEEP level within 3 d of being on ECMO was related to decreased mortality. Although no difference was observed in the PEEP levels between the two groups, we speculated that the down-regulation of PEEP during ECMO might have further aggravated the occurrence of collapse-induced injury, which led to atelectasis and sputum discharge obstacles. Therefore, the IPPV parameters, including high P_plat_ and VT levels and low PEEP settings, might have been unreasonable in our study; lung rest or the maintenance of open alveoli was not achieved.

The incidence of an ECMO oxygenator thrombus, haemorrhage, and organ failure in our study was high, which suggests that some problems existed in the anticoagulation management and organ supportive treatment of ECMO. We found that the unsuccessfully weaned group had larger fluctuations in ACT (the difference between ACT_max_ and ACT_min_ were larger) during the early stage of anticoagulation. This effect might suggest relatively unstable anticoagulation and a higher risk of haemorrhage. Moreover, the incidence rate of VAP during ECMO was as high as 60% and was partially attributed to the long course of H7N9 pneumonia and the prolonged duration of IPPV. Therefore, intensification of airway management was extremely necessary.

Our study had limitations. The nature of the study required the collection of data at multiple consecutive time points to evaluate the efficacy of ECMO. As a retrospective study with some missing data, we were unable to successfully collect data at 6 h pre-ECMO and 24, 48, and 72 h post-ECMO. Additionally, the number of subjects was too small to perform a multiple regression analysis to explore the risk factors for unsuccessful weaning from ECMO.

## Conclusions

ECMO is effective at improving oxygenation and ventilation of patients with avian influenza A (H7N9)-induced severe ARDS. Early initiation of ECMO with appropriate IPPV settings and anticoagulation strategies are necessary to reduce complications.

## Additional files

Additional file 1: Blood flow during ECMO, changes in IPPV parameters and physiological indicators pre-ECMO and during ECMO. (DOCX 107 kb)

Additional file 2: Blood flow during ECMO between the two groups. In the successfully weaned group vs. the unsuccessfully weaned group, a significant decrease in ECMO blood flow correlated with an increase in the duration of support, which was 3.65 ± 0.70 L/min vs. 4.57 ± 1.02 L/min, respectively, at 72 h (*P* < 0.05) and 3.65 ± 0.86 L/min vs. 4.62 ± 0.90 L/min, respectively, at 96 h (*P* < 0.01). (TIFF 185 kb)

Additional file 3: Changes in vital signs pre-ECMO and during ECMO between the two groups. Vital signs were improved and did not significantly differ between the two groups during ECMO. (TIFF 204 kb)

Additional file 4: ECMO Cases Per Year for Each Hospital. (DOCX 60 kb)

## Abbreviations

ABG: Arterial blood gas analysis
ACT: Activated coagulation time
APTT: Activated partial thromboplastin time
ARDS: Acute respiratory distress syndrome
CPAP: Continuous positive airway pressure
CRBSI: Catheter-related blood stream infection
DIC: Disseminated intravascular coagulation
ECMO: Extracorporeal membrane oxygenation
ELSO: Extracorporeal Life Support Organization
FiO_2_: Fraction of inspiration
HFOV: High frequency oscillatory ventilation
IPPV: Invasive positive pressure ventilation
MAP: Mean arterial pressure
MV: Mechanical ventilation
NAI: Neuraminidase inhibitors
NPPV: Non-invasive positive pressure ventilation
PaCO_2_: Partial pressure of arterial carbon dioxide
PaO_2_: Partial pressure of arterial oxygen
PEEP: Positive end-expiratory pressure
PP: Prone position
P_plat_: Plateau pressure
RM: Recruitment manoeuvre
SOFA: Sequential Organ Failure Assessment
SpO_2_: Fingertip pulse oxygen saturation
VAP: Ventilation-associated pneumonia
VILI: Ventilation-induced lung injury
VT: Tidal volume
