# Supplementary material for: Application of extracorporeal membrane oxygenation in patients with severe acute respiratory distress syndrome induced by avian influenza A (H7N9) viral pneumonia: national data from the Chinese multicentre collaboration
Source: BMC Infect Dis. 2018 Jan 8;18:23. doi: 10.1186/s12879-017-2903-x (PMC5759204; doi:10.1186/s12879-017-2903-x)
Supplement: Supplementary file 1 — Blood flow during ECMO, changes in IPPV parameters and physiological indicators pre-ECMO and during ECMO. (DOCX 107 kb) [file 12879_2017_2903_MOESM1_ESM.docx]

**Additional file 1. Blood flow on ECMO, changes in IPPV parameters and physiological indicators pre and on ECMO**

|  | Blood flow of ECMO (L/min) | | | FiO_2_ | | | PEEP (mmHg) | | | | |
| --- | --- | --- | --- | --- | --- | --- | --- | --- | --- | --- | --- |
|  | successfully  weaned | unsuccessfully  weaned | P value | successfully  weaned | unsuccessfully  weaned | P value | successfully  weaned | | unsuccessfully  weaned | | P value |
| 6h pre-ECMO | - | | | 92±16 | 96±11 | 0.481 | 14±4 | | 13±3 | | 0.552 |
| 24h on ECMO | 4.01±0.23 | 3.73±0.93 | 0.409 | 54±20 | 75±22 | 0.009* | 12±4 | | 11±4 | | 0.659 |
| 48h on ECMO | 4.13±0.70 | 4.67±0.75 | 0.073 | 46±13 | 74±25 | < 0.001* | 11±2 | | 12±3 | | 0.434 |
| 72h on ECMO | 3.65±0.70 | 4.57±1.02 | 0.016* | 45±11 | 78±24 | < 0.001* | 10±3 | | 11±2 | | 0.324 |
| 96h on ECMO | 3.65±0.86 | 4.62±0.90 | 0.009* | - | | | | | | | |
|  | VT (ml) | | | Pplat (mmHg) | | | Respiratory rate (/min) | | | | |
|  | successfully  weaned | unsuccessfully  weaned | P value | successfully  weaned | unsuccessfully  weaned | P value | successfully  weaned | unsuccessfully  weaned | | P value | |
| 6h pre-ECMO | 438±56 | 426±66 | 0.659 | 29±6 | 29±9 | 0.890 | 29±7 | 33±6 | | 0.095 | |
| 24h on ECMO | 244±94 | 304±91 | 0.164 | 22±4 | 24±7 | 0.392 | 19±5 | 20±6 | | 0.624 | |
| 48h on ECMO | 246±93 | 343±96 | 0.044* | 21±3 | 25±5 | 0.048* | 16±4 | 16±6 | | 0.867 | |
| 72h on ECMO | 236±113 | 356±116 | 0.037* | 19±4 | 29±6 | < 0.001* | 16±4 | 17±7 | | 0.719 | |
|  | Heart rate (/min) | | | MAP (mmHg) | | | SpO_2_ (%) | | | | |
|  | successfully  weaned | unsuccessfully  weaned | P value | successfully  weaned | unsuccessfully  weaned | P value | successfully  weaned | unsuccessfully  weaned | | P value | |
| 6h pre-ECMO | 100±16 | 105±33 | 0.674 | 91±14 | 80±15 | 0.050 | 76±13 | 75±14 | | 0.864 | |
| 24h on ECMO | 82±16 | 99±26 | 0.057 | 91±12 | 85±17 | 0.311 | 94±6 | 96±4 | | 0.559 | |
| 48h on ECMO | 80±14 | 88±25 | 0.323 | 81±14 | 81±25 | 0.991 | 95±5 | 93±6 | | 0.469 | |
| 72h on ECMO | 84±21 | 96±27 | 0.199 | 94±12 | 94±21 | 0.951 | 92±14 | 86±24 | | 0.488 | |
|  | pH | | | PaCO_2_ (mmHg) | | | PaO_2_ (mmHg) | | | | |
|  | successfully  weaned | unsuccessfully  weaned | P value | successfully  weaned | unsuccessfully  weaned | P value | successfully  weaned | unsuccessfully  weaned | | P value | |
| 6h pre-ECMO | 7.40±0.05 | 7.29±0.14 | 0.006* | 43.6±13.0 | 57.0±16.7 | 0.027* | 60.4±28.3 | 53.4±13.8 | | 0.386 | |
| 24h on ECMO | 7.44±0.07 | 7.41±0.10 | 0.455 | 37.1±14.2 | 32.5±6.1 | 0.263 | 110.8±37.1 | 88.3±33.6 | | 0.113 | |
| 48h on ECMO | 7.42±0.08 | 7.39±0.17 | 0.643 | 42.8±12.0 | 35.6±8.8 | 0.085 | 119.8±49.3 | 79.5±36.8 | | 0.018* | |
| 72h on ECMO | 7.44±0.07 | 7.43±0.10 | 0.813 | 40.5±7.1 | 36.9±8.2 | 0.285 | 141.5±65.3 | 81.1±38.0 | | 0.006* | |
|  | Lactate (mmol/L) | | |  | | | | | | | |
|  | successfully  weaned | unsuccessfully  weaned | P value |  |  |  |  |  |  |  |  |
| 6h pre-ECMO | 1.7±0.8 | 3.6±2.1 | 0.021* |  |  |  |  |  |  |  |  |
| 24h on ECMO | 1.5±0.5 | 4.1±1.1 | 0.031* |  |  |  |  |  |  |  |  |
| 48h on ECMO | 1.5±0.5 | 5.6±1.4 | 0.039* |  |  |  |  |  |  |  |  |
| 72h on ECMO | 1.6±0.4 | 4.0±3.4 | 0.047* |  |  |  |  |  |  |  |  |

Note: ECMO, extracorporeal membrane oxygenation; FiO_2_, fraction of inspiration；PEEP, positive end-expiratory pressure；VT, tidal volume; Pplat, plateau Pressure; MAP, mean arterial pressure; SpO_2_, fingertip pulse oxygen saturation；PaCO_2_, partial pressure of arterial carbon dioxide; PaO_2_, partial pressure of arterial oxygen. *, P<0.05.
