# Supplementary figures and images for: Application of extracorporeal membrane oxygenation in patients with severe acute respiratory distress syndrome induced by avian influenza A (H7N9) viral pneumonia: national data from the Chinese multicentre collaboration
Source: BMC Infect Dis. 2018 Jan 8;18:23. doi: 10.1186/s12879-017-2903-x (PMC5759204; doi:10.1186/s12879-017-2903-x)

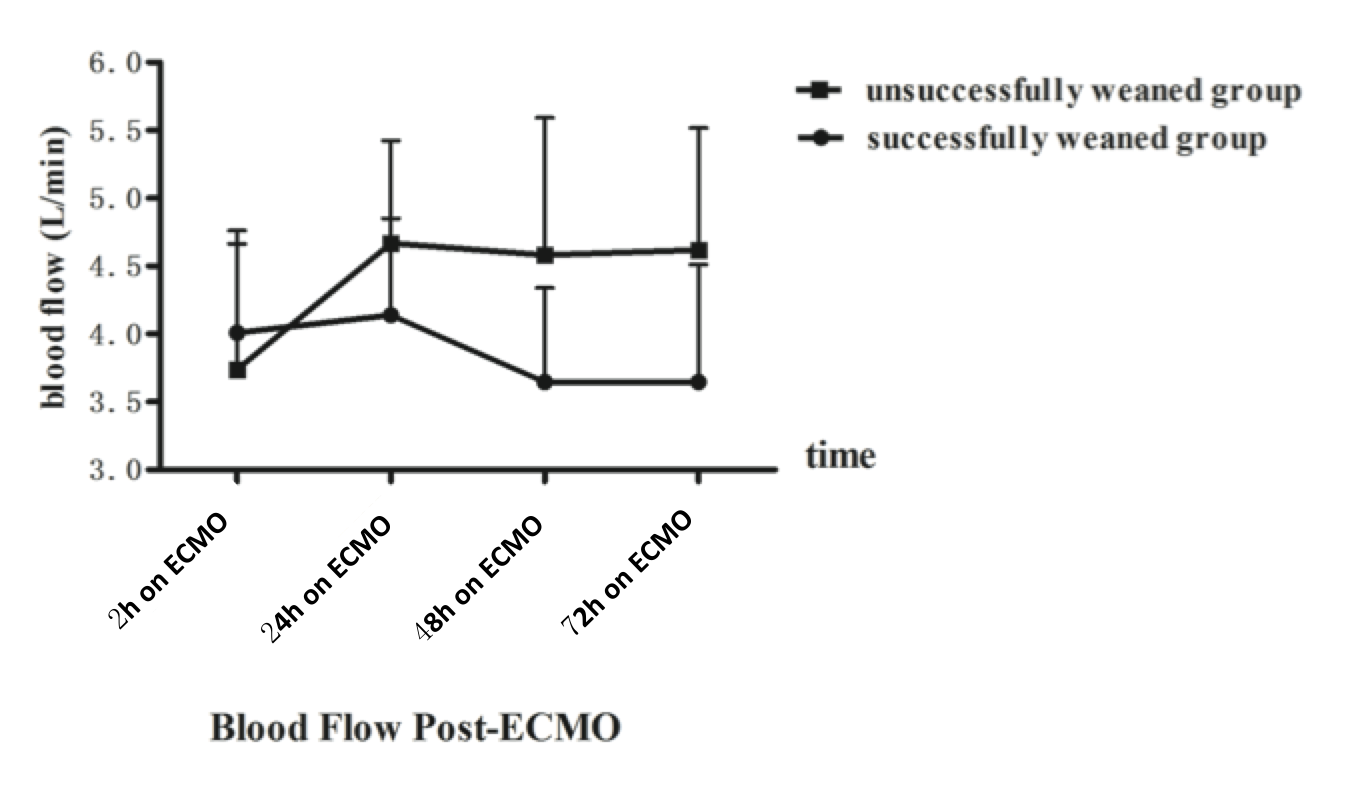

Supplement: Supplementary file 2 — Blood flow during ECMO between the two groups. In the successfully weaned group vs. the unsuccessfully weaned group, a significant decrease in ECMO blood flow correlated with an increase in the duration of support, which was 3.65 ± 0.70 L/min vs. 4.57 ± 1.02 L/min, respectively, at 72 h (P < 0.05) and 3.65 ± 0.86 L/min vs. 4.62 ± 0.90 L/min, respectively, at 96 h (P < 0.01). (TIFF 185 kb) [file 12879_2017_2903_MOESM2_ESM.tiff]

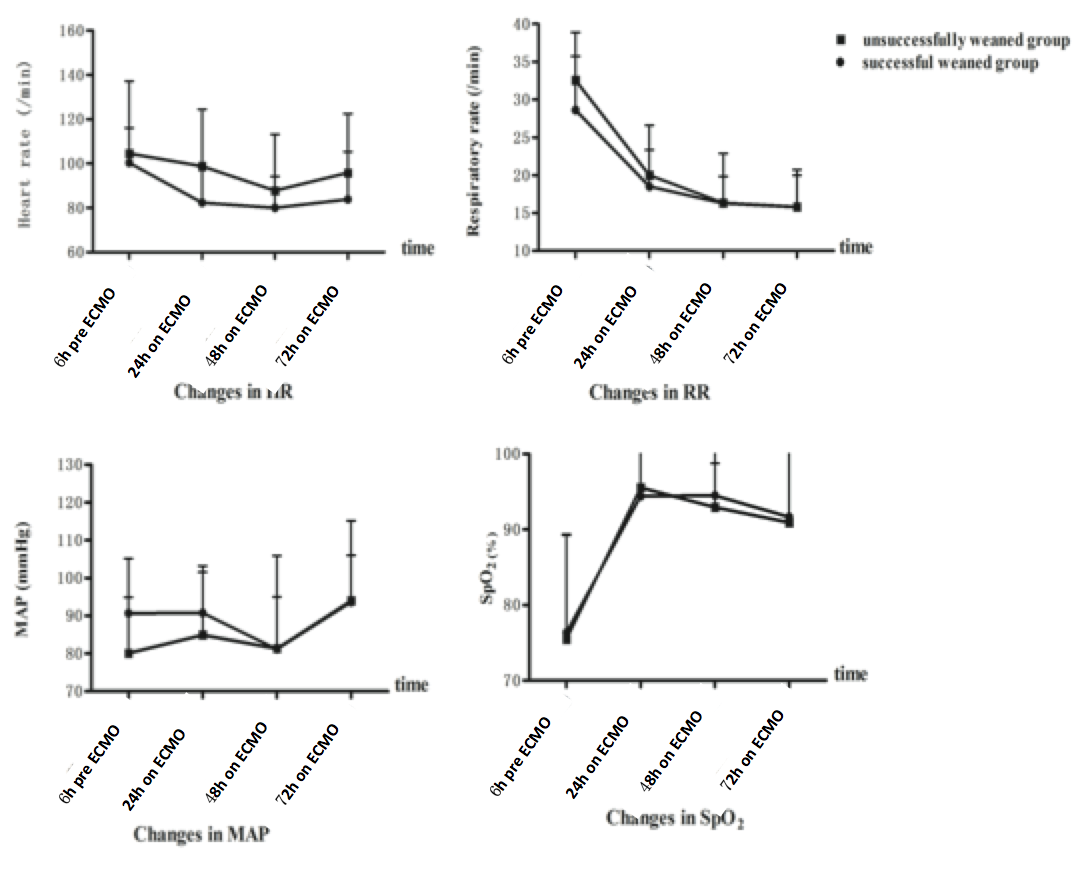

Supplement: Supplementary file 3 — Changes in vital signs pre-ECMO and during ECMO between the two groups. Vital signs were improved and did not significantly differ between the two groups during ECMO. (TIFF 204 kb) [file 12879_2017_2903_MOESM3_ESM.tiff]
