# Supplementary material for: Application of extracorporeal membrane oxygenation in patients with severe acute respiratory distress syndrome induced by avian influenza A (H7N9) viral pneumonia: national data from the Chinese multicentre collaboration
Source: BMC Infect Dis. 2018 Jan 8;18:23. doi: 10.1186/s12879-017-2903-x (PMC5759204; doi:10.1186/s12879-017-2903-x)
Supplement: Supplementary file 4 — ECMO Cases Per Year for Each Hospital. (DOCX 60 kb) [file 12879_2017_2903_MOESM4_ESM.docx]

**Additional File 4. ECMO Cases Per Year for Each Hospital**

| Hospitals | Cases of ECMO per year |
| --- | --- |
| The First Affiliated Hospital of Nanchang University, Jiangxi Province | 19 |
| Zhongda Hospital, Southeast University, Jiangsu Province | 25-30 |
| Wuhan Medical Treatment Centre Hospital, Hubei Province | 12 |
| The First Affiliated Hospital of Wannan Medical College, Yijishan Hospital, Wuhu, Anhui Province | 6 |
| Taizhou People's Hospital, Jiangsu Province, P. R. China | 4 |
| Affiliated Hospital of Nantong University, Jiangsu Province | 5 |
| Suzhou Municipal Hospital, Jiangsu Province | 5 |
| Henan Provincial People's Hospital, Henan Province | 5-10 |
| Dongguan People’s Hospital, Guangdong Province | 11 |
| The First People's Hospital of Kunshan, Jiangsu Province | 1 |
